# Mechanisms of Tolerance Induction in Liver Transplantation: Lessons Learned from Fetomaternal Tolerance, Autoimmunity and Tumor Immunity

**DOI:** 10.3390/ijms25179331

**Published:** 2024-08-28

**Authors:** Toshiaki Nakano, Shigeru Goto, Chao-Long Chen

**Affiliations:** 1 Graduate Institute of Clinical Medical Sciences, Chang Gung University College of Medicine, Taoyuan 333, Taiwan; 2 Liver Transplantation Center and Department of Surgery, Kaohsiung Chang Gung Memorial Hospital, Kaohsiung 833, Taiwan; pochigoto0224@gmail.com; 3 Nobeoka Medical Check Center, Fukuoka Institution of Occupational Health, Nobeoka 882-0872, Japan; 4 School of Pharmacy, Shujitsu University, Okayama 703-8516, Japan

**Keywords:** transplantation, post-transplant complications, tolerance, biomarkers, fetomaternal tolerance, autoimmunity, tumor immunity

## Abstract

Since the first published report of experimental kidney transplantation in dogs in 1902, there were many experimental and clinical trials of organ transplantation, with many sacrifices. After the establishment of the surgical technique and the discovery of immunosuppressive drugs, transplantation became the definitive treatment strategy for patients with terminal organ failure. However, this is not a common therapy method due to the difficulty of solving the fundamental issues behind organ transplantation, including the shortage of donor graft, potential risks of transplant surgery and economic capability. The pre- and post-transplant management of recipients is another critical issue that may affect transplant outcome. Most liver transplant recipients experience post-transplant complications, including infection, acute/chronic rejection, metabolic syndrome and the recurrence of hepatocellular carcinoma. Therefore, the early prediction and diagnosis of these complications may improve overall and disease-free survival. Furthermore, how to induce operational tolerance is the key to achieving the ultimate goal of transplantation. In this review, we focus on liver transplantation, which is known to achieve operational tolerance in some circumstances, and the mechanical similarities and differences between liver transplant immunology and fetomaternal tolerance, autoimmunity or tumor immunity are discussed.

## 1. History of Organ Transplantation

Organ transplantation is the ultimate treatment strategy to cure patients with terminal organ failure. Emerich Ullmann (1861–1937) was the pioneer surgeon who performed kidney transplantation in dogs in 1902 [1,2,3]. Although his first clinical trial of kidney xenotransplantation (pig to human) in the same year was not successful, it is no doubt that his innovative and challenging works led to the development of transplantation medicine. The first successful kidney transplantation was performed between identical twins by Joseph Murray (1919–2012) in 1954 [4]. However, allogeneic and xenogeneic transplantation generally failed due to the occurrence of graft rejection, the concept of which was demonstrated by Sir Peter Medawar (1915–1987), the father of transplantation [5,6].

In addition to the establishment of the surgical technique, the development of immunosuppressive drugs has dramatically changed the clinical application of organ transplantation. Azathioprine was the first immunosuppressive drug applied for kidney transplantation by Joseph Murray in 1962 [4]. The currently used immunosuppressive drug, cyclosporine, was identified from *Tolypocladiuminflatum* in 1969 [7], and its suppression of rejection after kidney transplantation was clinically demonstrated by Sir Roy Calne (1930–2024) in 1978 [8]. Another clinically used drug, tacrolimus, was isolated from *Streptomyces tsukubensis* in 1987 [9], and its clinical impact on the regulation of rejection in liver, kidney, pancreas, heart and lung transplantation was demonstrated by Thomas Starzl (1926–2017) in the late 1980s to the early 1990s [10,11].

Although these drugs effectively regulate post-transplant rejection, there are several limitations, including the necessity of long-term medication and the risk of adverse effects (e.g., nephrotoxicity, cardiovascular toxicity, cancer progression). Therefore, the goal of immunosuppressive therapy in transplantation is to find the optimal condition of immunosuppression for allograft survival by preventing rejection while limiting drug toxicities [12]. In order to achieve this goal, we need to carefully monitor pathophysiological and immunological conditions. Specifically, the ultimate goal should be the induction of operational tolerance after transplantation. In this review, we would like to focus on orthotopic liver transplantation (OLT), which is known to achieve operational tolerance in some circumstances, and the mechanical similarities and differences between liver transplant immunology and fetomaternal tolerance, autoimmunity or tumor immunity are discussed.

## 2. The Liver as an Immune-Privileged Organ

The liver is one of organs exposed to many endogenous and exogenous antigens, including food antigens and systemic infectious pathogens [13]. Therefore, the liver possesses unique immune system privileges for induced tolerance to harmless antigens, resulting in the maintenance of liver homeostasis. In addition, the prolongation of liver allograft survival without the need for immunosuppression was reported in pigs (donor: Landrace, recipient: Large White) [14,15]. However, the immunological aspects of liver transplant tolerance were not clearly described in the initial stage of transplant immunology research. This breakthrough could be seen after the introduction of the surgical technique for OLT in inbred rats with identical major histocompatibility complex (MHC) haplotypes. Briefly, Kamada and Calne published a remarkable paper in 1979introducing cuff methods for bile duct, lower vena cava and portal vein without anastomosing hepatic artery, leading to a shrinkage in the time of the anhepatic phase (less than 25 min) [16]. The application of Kamada’s cuff technique could increase the success rate of rat OLT (95.3% long-term survival) [17], and it opened the door to solving the immunological mysteries of liver transplantation. To be more specific, a fully allogeneic liver allograft can be spontaneously accepted in certain combinations [18] (Table 1), but other grafts (e.g., skin, kidney, heart) cannot. Furthermore, liver allografts induce donor-specific unresponsiveness, resulting in the acceptance of other grafts after OLT [19,20,21]. Although the mechanism of action of donor-specific tolerance induction has not been fully elucidated, the failure of tolerance induction by donor irradiation suggests the involvement of radiosensitive hepatic cells, including passenger leukocytes in liver transplant tolerance [22,23].

## 3. Impact of Liver Resident Cells on Liver Transplant Immunology

The liver possesses unique immune profiles as compared with those in the peripheral blood. For example, natural killer (NK) cells, NKT cells and γδ T cells are enriched in the liver [24]. In addition, a variety of hepatic antigen-presenting cells (APCs) such as hepatic dendritic cells (DCs), Kupffer cells, hepatic stellate cells and liver sinusoidal endothelial cells (LSECs) may play important roles in liver transplant rejection and tolerance [25]. The controversial roles of liver resident cells in inflammation/rejection and immune regulation/tolerance induction are summarized in Table 2.

### 3.1. Impact of Hepatic Innate-like Lymphocytes on Liver Transplant Immunology

NK cells play an important role in the detection and clearance of abnormal cells such as virus-infected cells, senescence cells and tumor cells. There are multiple roles of immune activation and inhibition in NK cells, which are dependent on the expression profiles of activating [natural killer group (NKG) 2D, natural cytotoxicity-triggering receptor 1 (NCR1) also known as NKp46] or inhibitory [NKG2A, killer cell immunoglobulin-like receptor (KIR)] receptors [26]. In addition, the expression levels of CD56 and CD16 can divide into two functionally different NK cell subpopulations such as CD56^dim^CD16^+^ cytotoxic NK cells, the most abundant NK cells in the peripheral blood, and CD56^blight^CD16^−^ NK cells, which possess weak cytotoxic activity [26]. In the case of liver transplantation in both animals and humans, donor-derived hepatic NK cells and recipient-derived peripheral blood NK cells may compete against each immune response, resulting in the orchestration of liver transplant rejection or tolerance [27,28]. In the course of rejection, NKG2D and its ligands (RAE1L and RRLT) were increased in rejected liver allografts in rats, which may reflect the infiltration of recipient peripheral blood NK cells with strong cytotoxic activity [29]. A peripheral NK cell shift to the liver graft was also observed in patients with early allograft dysfunction after liver transplantation, and NKG2D-mediated NK cell activation is a potential therapeutic target [30]. On the other hand, the transfusion of donor-type liver NK cells immediately after OLT alleviates liver allograft rejection in rats [31]. Furthermore, a recent study demonstrated a potential mechanism of action of donor liver-resident CD56^bright^CD16^+/−^ NK cells for the inhibition of recipient CD8^+^ T cell-mediated rejection in clinical liver transplantation [32].

NKT cells play a key role in the pathogenesis of liver diseases [33], and the impact of NKT cells on liver transplant rejection or tolerance is dependent on the different NKT cell subsets [34]. Type 1 NKT cells, also known as invariant NKT cells (iNKT cells), can recognize both host and microbial glycolipid antigens, and they rapidly release Th1 [e.g., interferon (IFN)-γ, tumor necrosis factor (TNF)-α], Th2 (e.g., interleukin (IL)-4, IL-10) and Th17 (e.g., IL-17, IL-22)-type cytokines upon activation [33]. Therefore, iNKT cells are subdivided into NKT1, NKT2 and NKT17 cells. Furthermore, IL-10-producing NKT10 cells are identified as a distinct regulatory iNKT cell subset in mice [35]. The involvement of iNKT cells in liver transplant tolerance has been demonstrated using iNKT-deficient mice [36]. In addition, the enhancement of iNKT cell activity by α-galactosylceramide (α-GalCer) could prolong liver allograft survival in rats, resulting in the suppression of IFN-γ but the elevation of IL-10 in the liver allograft [37]. On the other hand, Type 2 NKT cells, also known as diverse NKT cells (dNKT cells), have diverse T cell receptors, and they are more frequentthan iNKT cells in the mouse and human liver [38]. iNKT cells and dNKT cells can play an opposite role in liver inflammation. Briefly, iNKT cells were rapidly activated in the inflamed liver, while sulfatide-activated dNKT cells prevented liver inflammation through the induction of anergy in iNKT cells in mice [39]. On the other hand, sulfatide-reactive dNKT cells were increased in the liver, and they displayed a proinflammatory cytokine profile in patients with autoimmune hepatitis (AIH) [40]. Although the roles of dNKT cells in liver transplant rejection and tolerance are not fully elucidated, the balance of iNKT cells and dNKT cells in the liver may be one of fundamental issues for maintaining liver transplant tolerance.

γδ T cells are innate-type lymphocytes, and there are several subpopulations in humans according to their δ chain expression such as Vδ1^+^, Vδ2^+^ and Vδ3^+^ [41]. Vδ2^+^ γδ T cells are predominant in the peripheral blood and are mainly responsible for protecting the host from infection [42]. On the other hand, Vδ1^+^ γδ T cells are mainly located in the gut epithelia, dermis, spleen and liver [41]. Vδ3^+^ γδ T cells are mainly present in the liver and small intestinal epithelium [43]. Although there are no studies exploring the impact of Vδ3^+^ γδ T cells on liver transplant rejection and tolerance, the frequency of Vδ1^+^ and Vδ2^+^ γδ T cells in the liver allograft or in the peripheral blood has been proposed as a potential biomarker for prognosis in clinical liver transplantation. Briefly, the intragraft level of Vδ1^+^γδ T cells was elevated in recipients who accepted liver grafts with less immunosuppression or without immunosuppression after semiallogeneic pediatric OLT [44]. Another cohort study demonstrated the lower frequency of Vδ2^+^ γδ T cells in the peripheral blood of the recipients with stable liver allograft function, and the lower value of the Vδ1^+^/Vδ2^+^ ratio in peripheral blood may predict the risk of acute rejection [45].

### 3.2. Impact of Hepatic DCs on Liver Transplant Immunology

Hepatic DCs play an important role in host defense mechanisms to infectious pathogens but they skew toward an immune tolerance for harmless antigens such as food antigens and autoantigens [46]. Therefore, hepatic DCs may orchestrate the fate of liver transplant rejection or tolerance. In addition, the origins of hepatic DCs (donor-derived hepatic DCs or graft-infiltrating host DCs) may affect the post-transplant outcomes [47]. There are three distinct subsets of DCs in the liver, including myeloid/conventional DCs (cDCs), plasmacytoid DCs (pDCs) and monocyte-derived DCs (Mo-DCs) [48]. Among these subsets, Mo-DCs are derived from recipient bone marrow cells, and they are mainly associated with infection, inflammation or rejection after OLT [48]. Therefore, we discuss the impact of donor liver-resident hepatic cDCs and pDCs on liver transplant rejection and tolerance.

cDCs are derived from the common myeloid progenitor, and they are further divided into two subsets, cDC1 [Lineage (Lin)^−^ MHC II^+^ CD11c^+^ CD103^+^ CD11b^−^ in mice, Lin^−^human leukocyte antigen (HLA)-DR^+^ CD141^+^ XC-chemokine receptor 1(XCR1)^+^ C-type lectin domain containing 9A (CLEC9A)^+^ B- and T-lymphocyte attenuator 4 (BTLA4)^+^ in humans] and cDC2 [Lin^−^ MHC II^+^ CD11c^+^ CD103^−^ CD11b^+^ in mice, Lin^−^ HLA-DR^+^ CD1c^+^ CD1b^+^ CD14^+^ signal-regulatory protein alpha (SIRPα)^+^ in humans] [48]. cDC1 possess a superior ability to stimulate allogeneic and autologous naïve CD4^+^ T cells, as well as the induction of cytotoxic CD8^+^ T cells through cross-presentation for anti-viral and anti-tumor immune responses [49]. On the other hand, cDC2 present exogenous antigens to prime CD4^+^ T cell responses, and they play important roles in regulating type II immune responses to parasites, helminths and fungi through promoting Th2 responses and type 2 innate lymphoid cell activation [50,51]. In general, cDCs are believed to play an important role in liver transplant rejection [52]. In normal circumstances, however, hepatic cDCs maintain an immature state with a lower expression of MHC II and costimulatory molecules [53]. In addition, cDCs produce higher amounts of IL-10 in response to gut-derived bacterial lipopolysaccharide (LPS) [54]. Hepatic cDCs are less immunogenic than splenic cDCs, and the lower expression of Toll-like receptor 4 (TLR4) in hepatic cDCs may reduce the capacity to activate allogeneic T cells in response to LPS [55]. Taken together, there are controversial studies demonstrating the impact of cDCs on the induction of liver transplant rejection or tolerance. To be more specific, the fate of post-transplant outcomes may be dependent on the maturation state of hepatic cDCs [56].

pDCs [MHC II^int^ CD11c^int^ B220^+^ lymphocyte antigen 6 family C (Ly6C)^+^ bone marrow stromal cell antigen 2 (BST2)^+^ Sialic acid-binding immunoglobulin-like lectin H (Siglec-H)^+^ in mice and HLA-DR^+^CD11c^−^ CD4^+^ blood dendritic cell antigen (BDCA)2^+^ BDCA4^+^ CD123^+^ in humans] are derived from both myeloid and lymphoid progenitors, and they can rapidly secrete type I interferon in response to viral infection [47]. pDCs are predominant in the liver [57], and the administration of hepatic pDCs prolonged cardiac allografts [52,58]. Hepatic pDCs produced relatively higher levels of IL-10 as compared with splenic pDCs [59]. A lower expression of the Delta4/Jagged1 Notch ligand ratio in hepatic pDCs skewed towards Th2 cell differentiation and cytokine production, resulting in their inferior allostimulatory activity regulated by the presence and function of regulatory T cells (Treg) [59]. pDCs in the peripheral blood mononuclear cells (PBMCs) of operationally tolerant pediatric liver transplant recipients revealed a relatively higher expression of programmed death ligand-1 (PD-L1), and the ratio of pDCs and cDCs in PBMCs was significantly higher than other recipients under immunosuppression [60], thus suggesting the diagnostic potential of circulating the pDCs/cDCs ratio for the prediction of operational tolerance. A recent study using a mouse model of spontaneous liver transplant tolerance further demonstrated a potential mechanism of hepatic pDCs in the donor liver graft on the induction of liver transplant tolerance through a PD-L1-mediated reduction in graft-infiltrating CD8^+^ T cells with an exhausted phenotype [PD-1^+^, T cell immunoglobulin and mucin domain-containing protein 3 (TIM-3)] and the induction of Treg in the liver graft [61].

### 3.3. Impact of Kupffer Cells, Hepatic Stellate Cellsand LSECs on Liver Transplant Immunology

Kupffer cells are liver-resident macrophages located in the hepatic sinusoid where they phagocytize pathogens obtained from portal or arterial circulation [62]. Kupffer cells produce both proinflammatory and anti-inflammatory cytokines, and they may play a pivotal role in liver homeostasis. Earlier studies demonstrated the impact of gadolinium chloride, a Kupffer cell inhibitor, on the attenuation of liver injury [63,64,65]. Furthermore, gadolinium chloride could suppress acute rejection and lead to tolerance in a rat acute rejection (LEW liver into BN) model [66]. In addition to the impact of Kupffer cells on liver injury or liver transplant rejection, there are many studies regarding the involvement of Kupffer cells in portal venous tolerance induced by the intraportal injection of donor antigens [67,68,69]. Furthermore, the activation of Kupffer cells following OLT may play an important role in the induction of immune tolerance [70]. For example, overexpression of Fas ligand (FasL) in Kupffer cells induced allogeneic T cell apoptosis via the Fas/FasL pathway and regulated cytokine production such as IL-4, IL-10 and transforming growth factor (TGF)-β, resulting in the induction of liver transplant tolerance [71,72]. Induction of indoleamine 2,3-dioxygenase (IDO) in Kupffer cells may inhibit allogeneic T cell response followed by the induction of liver transplant tolerance in experimental animals [73,74,75]. A recent study demonstrated that overexpression of Rubicon, Beclin 1-interacting protein, in Kupffer cells induced LC3-associated phagocytosis (LAP) and the effective clearance of apoptotic allogeneic T cells, and polyunsaturated fatty acids (PUFAs), the product of apoptotic T cell degradation, activated peroxisome proliferator-activated receptor γ (PPARγ), resulting in the M2 polarization of Kupffer cells for the improvement of liver transplant outcome [76].

Hepatic stellate cells are located in the space of Disse between LSECs and hepatocytes, and they play pivotal roles in liver physiology and fibrogenesis [77]. The activation of hepatic stellate cells has been observed in chronically rejected liver allografts [78], and there are several risk factors for long-term liver allograft fibrosis including idiopathic post-transplant hepatitis and chronic antibody-mediated rejection [79]. On the other hand, the activation of hepatic stellate cells has important roles in the induction of immune tolerance via the induction of T cell apoptosis mediated by the Fas/FasL pathway and the stimulation of IL-10 and TGF-β production [80]. In addition, hepatic stellate cells directly inhibit T cells and B cells via PD-L1-mediated apoptosis induction [81,82]. Furthermore, the expression of CD44 in activated hepatic stellate cells not only promotes liver fibrosis [83] but also induces myeloid derived suppressor cells (MDSCs) from peripheral blood monocytes for immune modulation [84].

LSECs are the most abundant non-parenchymal cells, and they have important physiological and immunological functions including filtration, endocytosis, antigen presentation and lymphocyte recruitment for maintaining liver homeostasis [85]. LSECs express scavenger receptors (SRs) (e.g., SR-A1, SR-B1, SR-E1, SR-H1), IgG receptors [e.g., Fcγ receptor RIIb (FcγRIIb)] and TLRs (e.g., TLR1—4, 6, 8, 9) for the recognition and clearance of scavenger cells, antibody-coated immune complexes and pathogen-associated molecular patterns (PAMPs) or damage-associated molecular patterns (DAMPs), respectively [86]. Interestingly, LSECs in a normal condition are of a tolerogenic phenotype, and they play an important function in the induction of CD8^+^ T cell tolerance toward oral antigens (i.e., oral tolerance) [87]. In addition, there is much evidence regarding the impact of LSECs on the induction of allogeneic T cell tolerance across MHC barriers [88,89,90]. On the other hand, the induction of the pathological condition may shift the roles of LSECs from immune tolerance to inflammation. Aging is associated with the dysfunction of LSECs, resulting in the accumulation of neutrophils and macrophages and proinflammatory cytokine production in the liver [91]. The severe damage of LSECs during rejection after living donor liver transplantation has also been reported [92].

**Table 2:** Liver resident cells for the regulation of immune homeostasis.

Inflammation/Rejection	Immune Regulation/Tolerance Induction
Liver allograft Peripheral NKG2D^+^ CD56^dim^ CD16^+^ NK cells shift to the liver graft in the patients with EAD [30]	Liver allograft Donor liver-resident NKG2A^+^ CD56^bright^ CD16^+/−^ NK cells for the inhibition of CD8^+^ T cell-mediated rejection [32]
Inflamed liver Activation of iNKT cells during acute liver injury [39]; Proinflammatory cytokine production by sulfatide-reactive dNKT cells in patients with AIH [40]	Inflamed liver Prevention of liver inflammation by dNKT cells through the induction of anergy in iNKT cells [39] Liver allograft Tolerance induction by α-GalCer-activated iNKT cells [37]
Peripheral blood Lower value of the Vδ1^+^/Vδ2^+^ ratio as a predictive biomarker for rejection [45]	Liver allograft Elevation of Vδ1^+^γδ T cells in patients with stable liver allograft function [44]
Inflamed liver cDC1 for the stimulation of naïve CD4^+^ T cells and the induction of cytotoxic CD8^+^ T cells [49]; cDC2 for regulating type II immune responses to parasites, helminths and fungi [50,51]	Normal liver Immature state of cDCs (lower expression of MHC II and TLR4) with higher IL-10 production [53,54,55] Liver allograft PD-L1^+^pDCs-mediated reduction in infiltrating PD-1^+^ TIM-3^+^ CD8^+^ T cells and the induction of Treg [60,61]
Inflamed liver/Liver allograft Involvement of Kupffer cell activation in liver injury and liver transplant rejection [63,64,65,66]	Liver allograft Induction of FasL and IDO in Kupffer cells for the inhibition of alloreactive T cell response [71,72,73,74,75]
Liver allograft Involvement of hepatic stellate cell activation in long-term liver allograft fibrosis and chronic rejection [78,79]	Liver allograft Involvement of hepatic stellate cell activation in the induction of immune tolerance via Fas/FasL- or PD-L1-mediated apoptosis induction and MDSC induction [80,81,82,84]
Inflamed liver/Liver allograft Accumulation of neutrophils and macrophages and proinflammatory cytokine production in the liver by aging-associated LSEC dysfunction [91]; Rejection-induced severe damage of LSECs [92]	Normal liver LSEC-mediated induction of oral tolerance [87] Inflamed liver/Liver allograft LSEC-mediated clearance of scavenger cells, antibody-coated immune complexes and PAMPs/DAMPs [86]; Induction of allogeneic T cell tolerance [88,89,90]

NKG2D: natural killer group 2D, EAD: early allograft dysfunction, NKG2A: natural killer group 2A, iNKT cells: invariant NKT cells, dNKT cells: diverse NKT cells, AIH: autoimmune hepatitis, α-GalCer: α-galactosylceramide, cDCs: conventional DCs, PD-L1: programmed death ligand-1, pDCs: plasmacytoid DCs, TIM-3: T cell immunoglobulin and mucin domain-containing protein 3, Treg: regulatory T cells, FasL: Fas ligand, IDO: indoleamine 2,3-dioxygenase, MDSC: myeloid derived suppressor cells, LSEC: liver sinusoidal endothelial cells, PAMPs: pathogen-associated molecular patterns, DAMPs: damage-associated molecular patterns.

## 4. Mechanical Similarities and Differences between Liver Transplant Immunology and Fetomaternal Tolerance, Autoimmunity or Tumor Immunity 

Accumulating evidence (as shown below) suggests that there are several mechanical similarities between liver transplant immunology and fetomaternal tolerance for a successful pregnancy. Autoimmunity and allogeneic immune responses are, in general, similar pathological events, while these immune responses may regulate each other in certain circumstances. On the other hand, transplant immunology and tumor immunity function in the opposite fashion for host protection. The integration of knowledge from liver transplant immunology, fetomaternal tolerance, autoimmunity and tumor immunity may shed light on the mysterious immune system for maintaining immune homeostasis.

### 4.1. Liver Transplantation vs. Pregnancy

A successful pregnancy requires the induction of immune tolerance to a semi-allogeneic fetus, which contains paternal antigens (i.e., fetomaternal tolerance). Decidualization plays an important role in promoting placenta formation, and decidual stromal cells produce many factors required for the implantation and induction of fetomaternal tolerance [93]. During decidualization in early pregnancy, decidual stromal cells secret IL-24 and promote the differentiation of CD56^bright^ CD16^−^ NK cells in decidua [94]. The finding that donor liver-resident CD56^bright^ CD16^+/−^NK cells play an important role in the suppression of alloreactive T cell responses in liver transplantation [31] may suggest the involvement of decidual CD56^bright^ CD16^−^ NK cells in fetomaternal tolerance. The non-classical HLA class I molecules (HLA-G and HLA-E) are expressed in the human placenta, and they may interact with KIR receptors of NK cells for the maintenance of the pregnancy [95]. A recent meta-analysis revealed relatively lower soluble HLA-G expression in patients with pre-eclampsia as compared with patients having a normal pregnancy [96]. Similarly, higher HLA-G expression in serum and liver allograft is associated with a lower frequency of acute rejection in combined liver–kidney transplantation [97]. On the other hand, a higher percentage of Vδ2^+^γδ T cells and a lower percentage of Vδ1^+^γδ T cells in the peripheral blood were found in pregnant women at risk of premature pregnancy termination [98]. A similar reduction in the Vδ1^+^/Vδ2^+^ ratio in the peripheral blood has been reported as apredictive marker for acute rejection in liver transplantation [45], thus suggesting the existence of a common mechanism in CD56^bright^ CD16^−^ NK cells and Vδ1^+^γδ T cells for the induction of liver transplant tolerance and fetomaternal tolerance. However, the activation of decidual iNKT cells promotes pregnancy loss [99], while these cells in liver allografts play an important role in liver transplant tolerance [36,37]. A possible explanation for this opposite action of iNKT cells in liver transplantation and pregnancy may be the different immune systems between rodents and humans. Although the roles of dNKT cells in liver transplant rejection and tolerance are not fully elucidated, dNKT cells in decidua play a pivotal role in a successful pregnancy through the maintenance of Th2 environment [100]. Further studies including the characterization and functional assessment of innate-like lymphocytes are necessary for the complete understanding of liver transplant tolerance and fetomaternal tolerance.

In addition to innate-like lymphocytes, there are common mechanisms of tolerance induction induced by APCs in liver transplantation and pregnancy. For example, a balance between cDCs and pDCs is pivotal for the induction of fetomaternal tolerance. An IFN-γ-induced abnormal pregnancy model revealed the reduction in pDCs in decidua, accompanied by a lower Treg percentage in the para-aortic lymph nodesand decidua [101]. One of potential mechanisms of pDCs in the inhibition of rejection and subsequent tolerance induction is the expression of IDO in APCs [102]. Munn et al. first reported the prevention of allogeneic fetal rejection by IDO-mediated tryptophan catabolism, and IDO expression was confirmed in trophoblasts and macrophages [103]. In addition, a recent study demonstrated the expression of tryptophan-2,3-dioxygenase (TDO) co-expressed with IDO and Angiotensin (1–7) in placental tissues [104]. TDO is mainly expressed in the liver for tryptophan degradation in the kynurenine pathway [105]. In our previous study, we demonstrated the induction of IDO gene expression in the liver allograft during the rejection and tolerance induction phases in a rat tolerogenic OLT model (DA liver into PVG) [73]. Although little is known about the impact of Angiotensin (1–7) in liver transplant tolerance, Angiotensin (1–7) may play some roles in anti-inflammation and anti-fibrosis [106,107].

**Figure 1:**
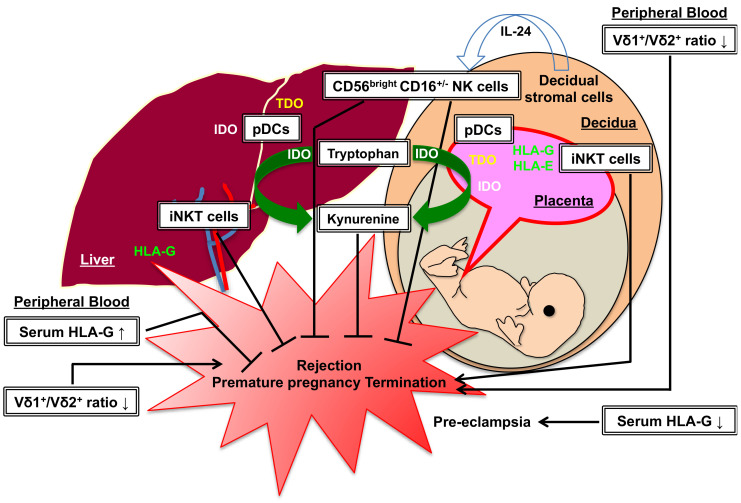
Mechanical similarities and differences between liver transplant immunology (rejection or tolerance) and pregnancy (abortion or fetomaternal tolerance). The induction of CD56^bright^CD16^+/−^ NK cells in the liver allograft or decidua may inhibit rejection or premature pregnancy termination. Hepatic or decidual APCs including pDCs express IDO and TDO for catabolizing tryptophan, which is required for T cell activation, resulting in the induction of liver allograft tolerance or fetomaternal tolerance through the kynurenine pathway. The higher expression of HLA-G/HLA-E in the placenta and the liver allograft is associated with immune tolerance. In the peripheral blood, the reduction in the Vδ1^+^/Vδ2^+^ ratio and the lower expression of HLA-G may be one of the predictive signatures for rejection, premature pregnancy termination or pre-eclampsia. On the other hand, the induction of iNKT cells in the liver allograft plays an important role in liver transplant tolerance. However, decidual iNKT cells promote abortion. APCs: antigen-presenting cells, pDCs: plasmacytoid dendritic cells, iNKT cells: invariant natural killer T cells, IDO: indoleamine-2,3-dioxygenase, TDO: tryptophan-2,3-dioxygenase, HLA: human leukocyte antigen, Vδ1^+^: Vδ1^+^γδ T cells, Vδ2^+^: Vδ2^+^γδ T cells.

Taken together, there are common mechanisms between rejection/abortion and tolerance induction in liver transplantation and pregnancy. The mechanical similarities and differences between liver transplant immunology and pregnancy are illustrated in Figure 1.

### 4.2. Liver Transplantation vs. Autoimmunity

Autoimmune liver diseases including AIH, primary biliary cholangitis (PBC) and primary sclerosing cholangitis (PSC) are chronic inflammatory hepatobiliary disorders leading to liver transplantation [108]. AIH is a T cell-mediated inflammatory disease, and major criteria for the diagnosis of AIH include the elevation of antinuclear antibody (ANA) and/or anti-smooth muscle antibody (ASMA) for type 1 AIH and the elevation of anti-liver-kidney microsomal type 1 antibody (ALKM-1) or anti-liver cytosol type 1 antibody (ALC-1) for type 2 AIH [109]. However, the fundamental roles of these autoantibodies in the pathogenesis of AIH are not fully understood [110]. In our previous studies, we demonstrated the elevation of nuclear antigens such as histone H1 and high mobility group box 1 (HMGB1) during the rejection phase after OLT [111] and how the transient elevation of corresponding ANA (anti-histone H1 antibody, anti-HMGB1 antibody) play an important role in overcoming rejection and the following tolerance induction both in experimental and clinical settings [112]. Therefore, extracellular release and/or exposure of nuclear antigens as alarmins may trigger liver transplant rejection or AIH, and the elevation of corresponding ANA may rather modulate unwanted immune responses. To support this hypothesis, we demonstrated the involvement of nuclear histone H1 as a therapeutic target in a rat model of concanavalin A (Con A)-induced AIH [113]. Some studies demonstrated the impact of HMGB1 signaling on the pathogenesis of AIH, and the blockade of HMGB1 signaling may ameliorate experimental AIH [114,115]. The elevation of ANA against histone H1 or HMGB1 in PBC patients [116,117] and the elevation of biliary HMGB1 in PSC patients [118] also suggest that histone H1 and HMGB1 may act as a potential therapeutic target in autoimmune liver diseases.

Treg are key players in the maintenance of immune homeostasis by preventing immune responses to self-antigens [119]. In AIH, CD39^+^ Treg are decreased and fail to hydrolyze proinflammatory nucleotides into immunosuppressive adenosine, resulting in the activation of Th17 cells [120]. In addition, the balance between Treg and Th17 cells (Treg/Th17 ratio) in PBMCs may predict disease severity in autoimmune liver diseases [121,122]. In liver transplantation, CD39 expression in liver allografts may modulate Treg infiltration for the suppression of rejection and tolerance induction [123]. The reduced expression of the Treg/Th17 ratio is also associated with liver transplant rejection [124,125]. Our previous study demonstrated the impact of hepatic miR-301a on the regulation of IL-6 production in hepatocytes and the induction of Th17 cell differentiation [126]. Although there is no direct evidence that hepatic miR-301a regulates autoimmune liver diseases, the elevation of miR-301a in PBMCs may serve as a pathogenic factor in systemic lupus erythematosus [127]. Therefore, hepatic and circulating miR-301a may act as a potential therapeutic target for the induction of immune tolerance in liver transplant immunology and autoimmunity.

In general, recurrent AIH or de novo AIH after liver transplantation may lead to graft dysfunction [128]. De novo AIH is also known as plasma cell-rich rejection or plasma cell hepatitis, and a recent physiological and pathological characterization revealed the presence of donor-specific antibodies (DSAs) and complement C4d in de novo AIH patients, which is within the histologic spectrum of antibody-mediated rejection [129]. In addition, liver transplant recipients with PBC or PSC have also been found to have an increased risk of early and late T cell-mediated rejection [130]. By contrast, the simultaneous induction of liver transplant rejection and de novo AIH by Con A injection after OLT could prolong liver allograft survival with the spontaneous elevation of ANA against histone H1 or HMGB1, resulting inreduction in DSA in experimental animals [131]. Another AIH model by α-GalCer administration through the caudal vein with OLT alsorevealed the prolongation of liver allograft survival [37]. Although experimental animals do not fully mimic the clinical manifestation of AIH, the transient elevation of ANA against nuclear antigens during the recovery phase after AIH may be one of the important phenomena for maintaining immune homeostasis [113]. Furthermore, the induction of AIH after OLT in experimental animals may modulate the balance between allogeneic immune responses and autoimmune responses during rejection, resulting in the acquisition of immune tolerance [131]. However, the continuous elevation of ANA may increase the risk of autoimmune diseases.

**Figure 2:**
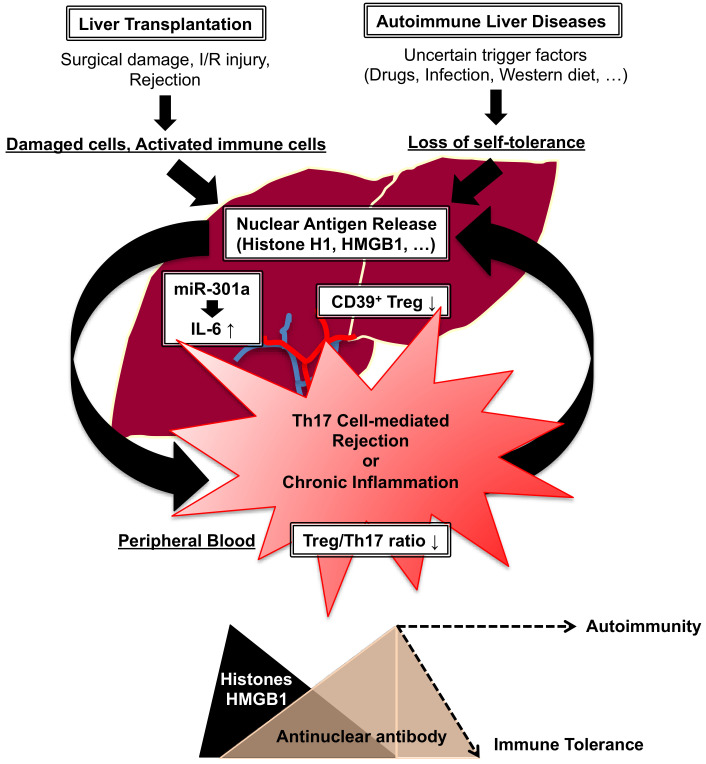
Mechanical similarities and differences between liver transplant immunology (rejection or tolerance) and autoimmune liver diseases. Although the trigger of nuclear antigen release is different, damaged cells and activated immune cells may release nuclear antigens such as histone H1 and HMGB1, resulting in the induction of rejection and inflammatory responses. Overexpression of hepatic miR-301a may lead to the production of IL-6 for the induction of Th17 cell differentiation during rejection and autoimmune liver diseases. The reduction in CD39^+^ Treg in the liver may be associated with liver transplant rejection and autoimmune liver diseases partly through the activation of Th17 cells. The reduction in the Treg/Th17 ratio has been proposed as a predictive marker for rejection or autoimmune liver diseases. In some circumstances, the transient induction of antinuclear antibody against histone H1 or HMGB1 may neutralize nuclear antigens, resulting in the induction of immune tolerance in experimental animals. However, the long-term elevation of antinuclear antibody may be associated with a loss of immune homeostasis. I/R injury: ischemia/reperfusion injury, HMGB1: high mobility group box 1, Treg: regulatory T cells.

Taken together, there are common mechanisms in the pathogenesis of liver transplant rejection and autoimmune liver diseases, and studies on experimental animals may give some hints for the induction of immune tolerance. Mechanical similarities and differences between liver transplant immunology and autoimmune liver diseases are illustrated in Figure 2.

### 4.3. Liver Transplantation vs. Tumor Immunity

Hepatocellular carcinoma (HCC) is the most common type of primary liver cancer, and the recurrence of HCC after surgical resection or liver transplantation is one of critical issues impacting mortality in HCC patients [132]. In general, transplant immunology and tumor immunity function in the opposite fashion for host protection. In other words, the mechanism of action of transplant rejection may be applicable for the activation of tumor immunity (tumor immune surveillance). In this review, we have focused on the impact of HCC-derived exosomal components, including microRNAs for immune modulation.

As shown in Table 3, several microRNAs were identified as predictive biomarkers for rejection in experimental and clinical liver transplantation. For example, liver transplant rejection-associated microRNAs (miR-146a, miR-199a-3p, miR-181a-5p and miR-122-5p) may act as tumor suppressor microRNAs through the suppression of vascular endothelial growth factor (VEGF), mammalian target for rapamycin (mTOR), p21-activated kinase 4 (PAK4), early growth response factor 1 (Egr1) and multidrug resistance protein 1 (MDR1), respectively [133,134,135,136]. On the other hand, overexpression of miR-301a and miR-155-5p in the liver may be associated with tumorigenesis through the inhibition of interferon regulatory factor-1 (IRF-1) and the induction of M2 macrophage polarization [137,138]. Therefore, we need to carefully monitor the intensity and combination of these microRNAs in order tomaintain good balance for the prevention of both rejection and HCC recurrence after liver transplantation.

**Figure 3:**
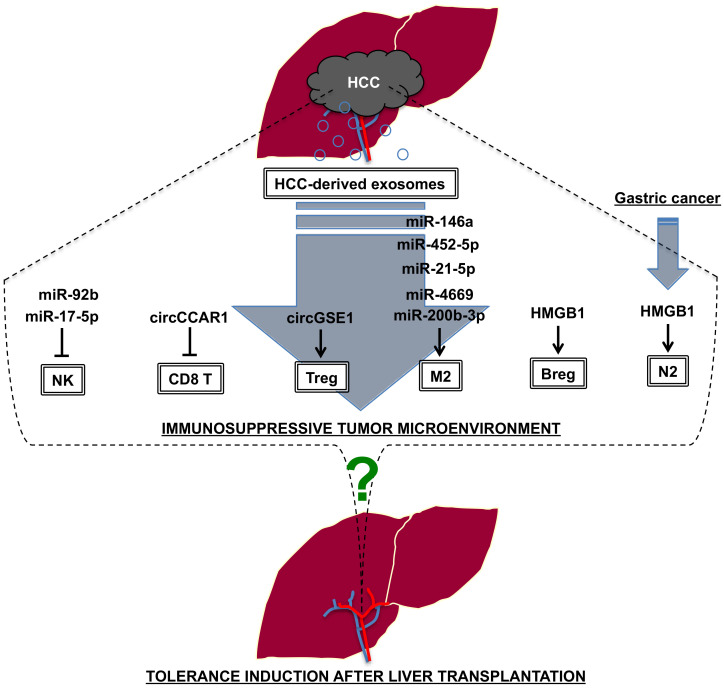
HCC-derived exosomes generate an immunosuppressive tumor microenvironment, but little is known whether these exosomal components can induce liver transplant tolerance. NK: natural killer cells, CD8 T: CD8 T cells, Treg: regulatory T cells, M2: M2 macrophages, Breg: regulatory B cells, N2: N2 neutrophils.

Conversely, understanding the mechanism of action of tumor immune escape induced by aggressive tumor cells may lead to a novel strategy for the induction of immune tolerance. There are many studies regarding the mechanism of action of immune escape in HCC. One of the potential mechanisms is the release of exosomalcomponents in HCC for the promotion of an immunosuppressive tumor microenvironment (Figure 3). In our previous study, we identified exosomal miR-92b as a predictive biomarker for HCC recurrence after OLT, partly through the downregulation of NK cell function by targeting CD69 [149]. HCC-derived exosomal miR-17-5p also downregulated NK cell function by targeting runt-related transcription factor 1 (RUNX1)-NKG2D axis [150]. On the other hand, CD8^+^ cytotoxic T cell activity was reduced by exosomal circular RNA circCCAR1 derived from HCC cells, and it induced resistance to anti-PD-1 immunotherapy [151]. For the induction of Treg in the tumor microenvironment, exosomal circGSE1 was released from HCC cells and regulated miR-324-5p/TGF-β receptor 1 (TGFBR1)/SMAD family member 3 (SMAD3) axis [152]. There are many exosomal microRNAs associated with the induction of M2 macrophage polarization in HCC, including miR-146a-5p [153], miR-452-5p [154], miR-21-5p [155], miR-4669 [156] and miR-200b-3p [157]. In addition to microRNAs, HCC-derived exosomes contain HMGB1, which promote T cell immunoglobulin and mucin domain-1 (TIM-1)^+^ regulatory B cell (Breg) expansion [158]. Tumor-derived exosomal HMGB1 also induces N2 polarization of neutrophils, resulting in the promotion of gastric cancer cell migration [159]. The potential impact of HCC-derived exosomes on the N2 polarization of neutrophils has also been reported in HCC [160]. So far, little is known about the impact of these exosomal components in liver transplant tolerance. Further investigation including comparative studies to demonstrate the common factors associated with tumor immune escape and liver transplant tolerance should be considered in the future.

## 5. Discussion

In this review, we have summarized the current understanding of liver transplant immunology, including characterization of unique immune and hepatic cell profiles and their functional roles in rejection and tolerance induction. There are many cellular and humoral factors associated with liver transplant rejection and tolerance. Interestingly, there are common mechanisms of rejection and tolerance between liver transplantation and pregnancy. Common mechanisms of rejection and autoimmune liver diseases and the unique phenomena of experimental animals in tolerance induction may give some hints for immune modulation. We also introduced the mechanisms of action of tumor immune escape in HCC, which may be applicable for the induction of immune tolerance in liver transplantation.

Briefly, the activation of CD56^bright^ CD16^+/−^ NK cells in the liver allograft or decidua may be one of common mechanisms for the induction of immune tolerance in liver transplantation [31] and successful pregnancy [94]. The recruitment of CD56^dim^CD16^+^ cytotoxic NK cells into the liver may contribute to AIH progression [161], and a similar phenomenon has been observed in liver transplant rejection [30] or pregnancy loss [162]. By contrast, HCC progression may modulate NK cell proportions, and patients with effective treatment response to sorafenib revealed a reduced ratio of CD56^bright^ and CD56^dim^ NK cells [163]. The higher expression of HLA-G may contribute to liver transplant tolerance [97] and successful pregnancy [95], while AIH patients, notably patients with severe liver inflammation, revealed significantly lower soluble HLA-G levels [164]. On the other hand, the aberrant expression of HLA-G has been observed in HCC patients and correlated with poor prognosis [165]. The higher expression of IDO in the liver allograft or placenta may be associated with liver transplant tolerance [105] and successful pregnancy [104]. On the other hand, IDO overexpression in HCC was significantly correlated with high metastasis rates and poor prognosis due to the promotion of tumor immune escape [166]. The proportion of CD39^+^ Treg in the liver may be associated with liver transplant tolerance [123] and self-tolerance [120]. By contrast, CD39^+^ Treg infiltration in HCC has been reported as an independent predictor for HCC aggressiveness and postoperative recurrence [167]. In summary, these findings point to the mechanical similarity and complications in immune regulation among liver transplant immunology, fetomaternal tolerance, autoimmunity and tumor immunity.

In the previous and current studies, researchers used experimental animals and clinical specimens to identify and characterize potential biomarkers for diagnosis and therapeutics in liver transplantation, pregnancy, autoimmune liver diseases or HCC, independently. Future research directions should be the integration of the knowledge obtained from liver transplant immunology, fetomaternal tolerance, autoimmunity or tumor immunity, which may narrow down the key factors for optimal immune modulation. In addition, we need to monitor many biomarkers such as microRNAs to understand the time- or condition-dependent regulation of these expression profiles in liver transplantation (rejection and tolerance), pregnancy (abortion and fetomaternal tolerance), autoimmunity (disease severity) and tumor immunity (tumor size, grade and metastasis).

## Figures and Tables

**Table 1:** Rat OLT moldels for transplant immunology research.

Donor (MHC)	Recipient (MHC)	Model	Reference
DA (RT1^a^)	PVG (RT1^c^)	Tolerance	[17]
PVG (RT1^c^)	DA (RT1^a^)	Tolerance	[18]
DA (RT1^a^)	LEW (RT1^l^)	Acute rejection	[18]
LEW (RT1^l^)	DA (RT1^a^)	Tolerance	[18]
BN (RT1^n^)	LEW (RT1^l^)	Tolerance (delayed rejection)	[18]
LEW (RT1^l^)	BN (RT1^n^)	Acute rejection	[18]

MHC: major histocompatibility complex, DA: Dark Agouti, PVG: Piebald Virol Glaxo, LEW: Lewis, BN: Brown Norway, RT1: rat MHC haplotype.

**Table 3:** Liver transplant rejection-associated microRNAs and their impact on HCC.

Rejection Marker	Expression	Functions in HCC	Reference
miR-146a (rat) [139]	Plasma, Liver tissue	Lower expression of miR-146a in HCC tissues (human)	[140]
		Tumor suppressor microRNA, VEGF inhibition (human, cell line)	[133]
miR-301a (rat) [126]	Liver tissue	Upregulation of miR-301a in HCC tissues (human)	[141]
		Onco-microRNA, IRF-1 inhibition (human, cell line)	[137]
miR-199a-3p (rat) [142]	Plasma, Liver tissue	Lower expression of miR-199a-3p in HCC tissues (human)	[143]
		Tumor suppressor microRNA, mTOR, PAK4 inhibition (animal model)	[134]
miR-181a-5p (human) [144]	Plasma	Lower expression of miR-181a-5p in HCC tissues (human)	[145]
		Tumor suppressor microRNA, Egr1 inhibition (human, cell line)	[135]
miR-155-5p (human) [144]	Plasma	Upregulation of miR-155-5p in HCC tissues (human)	[146]
		Onco-microRNA, M2 macrophage polarization (human)	[138]
miR-122-5p (human) [147]	Plasma	Lower expression of miR-122-5p in HCC tissues (human)	[148]
		Tumor suppressor microRNA, MDR1 inhibition (cell line, animal model)	[136]

VEGF: vascular endothelial growth factor, IRF-1: interferon regulatory factor-1, mTOR: mammalian target of rapamycin, PAK4: p21-activated kinase 4, Egr1: early growth response factor 1, MDR1: multidrug resistance protein 1.
